# ATPase Cycle and DNA Unwinding Kinetics of RecG Helicase

**DOI:** 10.1371/journal.pone.0038270

**Published:** 2012-06-06

**Authors:** Christopher P. Toseland, Ben Powell, Martin R. Webb

**Affiliations:** MRC National Institute for Medical Research, Mill Hill, London, United Kingdom; University of Massachusetts Medical School, United States of America

## Abstract

The superfamily 2 bacterial helicase, RecG, is a monomeric enzyme with a role in DNA repair by reversing stalled replication forks. The helicase must act specifically and rapidly to prevent replication fork collapse. We have shown that RecG binds tightly and rapidly to four-strand oligonucleotide junctions, which mimic a stalled replication fork. The helicase unwinds such DNA junctions with a step-size of approximately four bases per ATP hydrolyzed. To gain an insight into this mechanism, we used fluorescent stopped-flow and quenched-flow to measure individual steps within the ATPase cycle of RecG, when bound to a DNA junction. The fluorescent ATP analogue, mantATP, was used throughout to determine the rate limiting steps, effects due to DNA and the main states in the cycle. Measurements, when possible, were also performed with unlabeled ATP to confirm the mechanism. The data show that the chemical step of hydrolysis is the rate limiting step in the cycle and that this step is greatly accelerated by bound DNA. The ADP release rate is similar to the cleavage rate, so that bound ATP and ADP would be the main states during the ATP cycle. Evidence is provided that the main structural rearrangements, which bring about DNA unwinding, are linked to these states.

## Introduction

DNA helicases are motor proteins with essential roles in many aspects of DNA metabolism such as replication, recombination and repair. The chemical energy of nucleoside triphosphate hydrolysis, generally ATP, is used to drive the mechanical action of nucleic acid strand separation and translocation by the helicase. Work is presented here on RecG, a bacterial helicase from Superfamily 2 (SF2), which comprises the largest superfamily containing several subfamilies including DEAD-box RNA helicases [1], the RecQ-like family [2] and the Snf2-like enzymes [3], [4].

Bacterial DNA replication is only partially continuous and processive. Efficient replication is essential but DNA polymerase complexes often fail to complete that process, as they are hindered by numerous factors, such as lesions or proteins bound to DNA. This causes stalling, which can lead to replication fork collapse, and so creates free DNA ends, which can lead to genome rearrangements. Therefore, complete replication is dependent upon efficient repair to bypass or remove lesions and recombination events to resolve stalled replication forks [5]. One such mechanism is fork regression, which facilitates the removal of a lesion in one of the strands via the formation of a four-stranded DNA structure, called a Holliday junction. RecG catalyzes this process by unwinding newly replicated arms from the junction, annealing nascent strands and re-annealing parental strands. Subsequently, the Holliday junction can be migrated and resolved by RuvABC [6], [7], allowing replication to continue.

A single structure of RecG has been solved [8] but the DNA substrate was not long enough to interact with the motor domains. Thus, there is limited information about structural aspects of the translocation mechanism, or conformation changes that occur in the protein during the ATPase cycle. However, a scheme was proposed for linking duplex translocation from a DNA binding loop to the ATP binding site [9]. A greater understanding of the chemo-mechanical coupling of this helicase will allow this model to be more rigorously tested.

**Figure 1:**
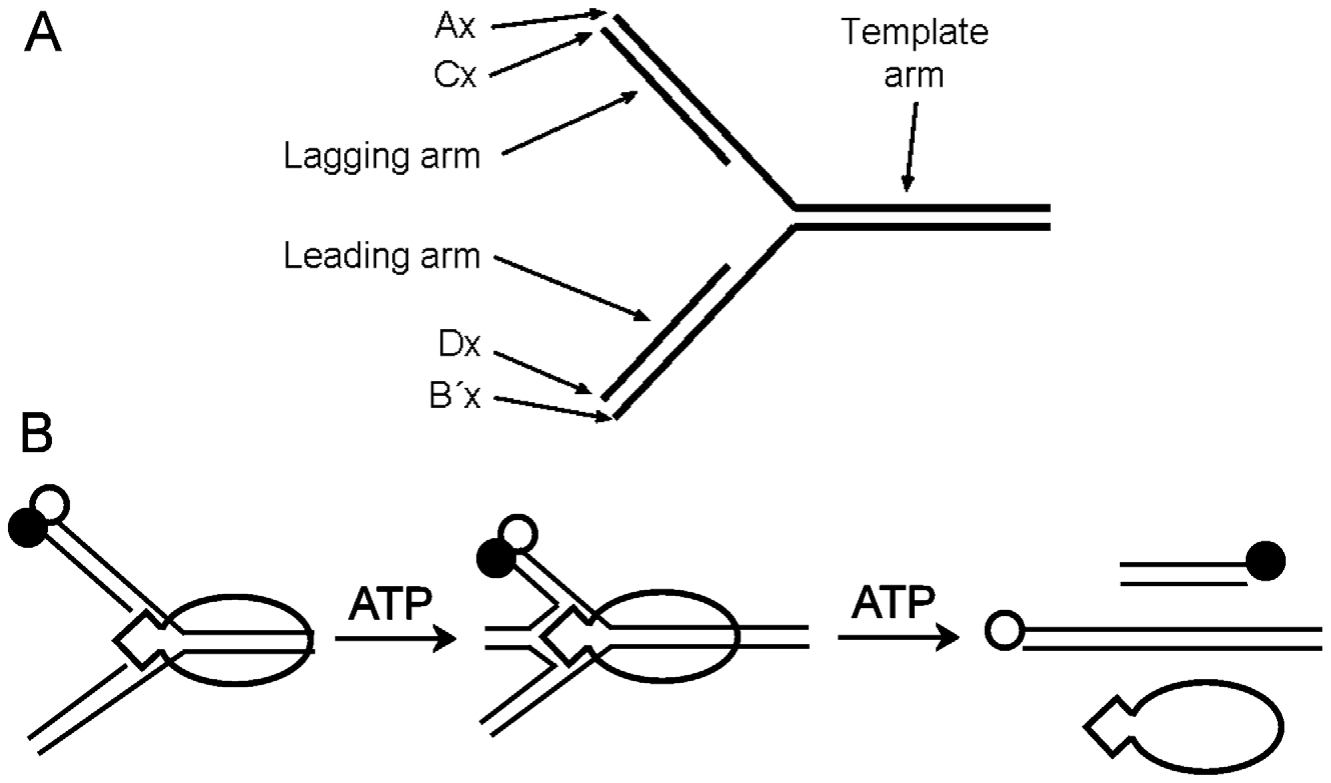
DNA junctions and unwinding scheme. (A) Schematic representation of a model DNA substrate. The template strand forms the Ax series, where x is the number of nucleotides. The leading strand forms the Bx, if non-complementary, or the B′x series, if complementary. The lagging strand forms the Cx series. The strand complementary to the leading strand forms the Dx series. (B) Cartoon scheme showing unwinding of a complementary DNA junction by RecG. This example has a 5′-Cy3 label (white circle) on the template strand and a 3′-Dabcyl on the lagging strand (black circle).

A kinetic analysis of the ATPase cycle for *Thermotoga maritima* RecG is presented here to determine the main intermediates during the cycle and what biochemical steps may be coupled to translocation. The ATPase cycle was investigated by measuring rate constants for individual processes in the cycle. The complete cycle was measured using the analogue mantATP ((2′(3′)-*O*-(*N*-methylanthraniloyl)ATP), to provide a consistent view of the cycle, as some steps cannot be measured with the natural ATP substrate. However, because the nucleotide modification caused changes in kinetics for some parts of the ATPase cycle, several key steps were measured with the natural substrate, so that the ATPase mechanism with the ATP analogue could be related to that with unlabeled nucleotide. In particular mantATP is hydrolytically cleaved ∼10-fold slower than ATP and the release of mantADP is very slow. Despite these differences, the fluorescent ATP allowed definition of an ATPase mechanism and how this might be related to structural changes. Finally, DNA unwinding and corresponding ATPase measurements were used to relate the ATP usage with translocation for an expanded range of model substrates. This included homologous sequence throughout to mimic a stalled replication fork (Figure 1). These substrates contain four oligonucleotides, so that partial unwinding produces a chicken-foot structure and on complete unwinding two separate duplexes result.

## Results

### Steady-state ATPase Rate Measurements

**Figure 2:**
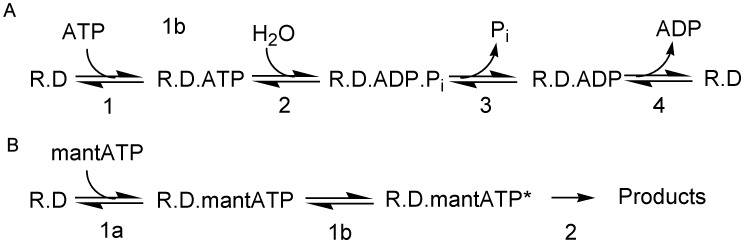
ATPase reaction schemes. (A) Minimal mechanism for ATP hydrolysis by RecG (R) with DNA (D). Steps are numbered, such that step *n* has forward and reverse rate constants, *k*_+n_ and *k*_-n_, respectively, and equilibrium constant, *K*_n_. (B) Scheme for two-step binding of mantATP.

Steady-state measurements were made to gain an overall assessment of the reaction and the affinities of various nucleotides before investigating the individual steps in the ATPase cycle. These steps are described by the basic scheme shown in Figure 2A. A two-strand DNA junction, which contains two 20-bp regions of complementary and non-complementary sequences (A40:B40 Figure 1 and Table S1), was used in all these measurements. This forms a Y-shape structure and RecG translocates one single-stranded arm, but then remains bound to the end of the junction still hydrolyzing ATP [10]. Use of this junction simplifies the system as the DNA substrate is unmodified by RecG action and more significantly, ssDNA is not produced.

In the absence of DNA, the ATPase activity was very low: DNA activates ATPase activity 50-fold (Table 1a). Figure S1A shows the rate of ATP hydrolysis as a function of nucleotide concentration in the presence of the DNA junction, giving a *K*_m_ value of 9 (±2) µM for ATP and a *k*_cat_ of 8.3 (±1.7) s^−1^. The values of *k*_cat_ and *K*_m_ differ from that previously reported with values of 5.2 s^−1^ and 42 µM, respectively, as the latter were measured at a much lower concentration of DNA [10], resulting in a lower concentration of RecG⋅DNA complexes. The fluorescent ATP analogue, mantATP gave *K*_m_ as 1.9 (±0.6) µM and *k*_cat_ 0.3 (±0.1) s^−1^ (Figure S1B), rather different values from those with ATP. The mantATP *k*_cat_ value is unlikely to be defined well due to inhibition resulting from the high affinity of mantADP (see below).

**Table 1:** Steady-state ATPase kinetics for RecG.

(a)		
Nucleotide	*k*_cat_ (s^−1^)	*K*_m_ (µM)
ATP	8.3 (±1.7)	9 (±2)
ATP (no DNA)	0.2 (±0.1)	7.5 (±3.1)
MantATP	0.3 (±0.1)	1.9 (±0.6)
Mant-deoxyATP	1.3 (±0.5)	3.1 (±1.4)
**(b)**		
**Nucleotide**	***K*** **_i_** (µM)	
ADP	7.3 (±2.1)	
AMPPNP	44 (±4.7)	
MantADP	0.03 (±0.01)	
Mant-deoxyADP	0.1 (±0.05)	
ATPγS	0.9 (±0.4)	

All measurements were carried out at 20°C in the presence 10 µM MDCC-PBP, 10 nM RecG and 500 nM DNA junction (A40:B40) in a buffer described in the materials and methods. (a) *k*_cat_ and *K*_m_ for nucleoside triphosphates (b) *K*_i_ for non-hydrolyzable or slowly hydrolyzing nucleotides, using 10 µM ATP and varying inhibitor concentration.

The steady state assay was also used to assess the tightness of binding of other nucleotides through competitive inhibition measurements (Table 1b). ADP binds quite tightly (*K*_i_ 7.3 (±2.1) µM), but the affinity of mantADP was over two orders of magnitude greater (*K*_i_ 30 (±10) nM). The “non-hydrolyzable” ATP analogue, AMPPNP bound weakly and so was not likely to be useful as a mimic of ATP in binding studies here. In contrast, another analogue, ATPγS bound tightly with an affinity similar to ATP, but was slowly hydrolyzed at a rate of 0.1 s^−1^. MantATP, and other fluorescent nucleotides modified at the ribose ring (data not shown) were all hydrolyzed more slowly than non-modified ATP. However, the fluorescence signal and high affinity did provide advantages in order to investigate the ATPase mechanism.

### Assessment of mantATP as a Substrate for RecG

In order to investigate the kinetics of the ATPase cycle, signals are required that report on each step. The scheme in Figure 2A shows a minimal ATPase cycle, expected to occur during translocation. RecG is assumed to remain bound to the DNA throughout: initial RecG binding to this DNA substrate was measured previously [10]. Fluorescent adenine nucleotides potentially provide signals for their binding to and release from proteins. While fluorescent labeling can perturb the affinity, mant nucleotides are frequently used and it is one of the smallest such modification available [11]. Use of the fluorescent nucleotide, albeit with changed kinetics, allows a model mechanism to be made and then tested with ATP itself. This would not be possible using ATP alone, as some steps give no signal and the weak nucleotide affinity precludes measurements requiring quantitative complex formation. Although signals are not available for all processes, some steps can be measured with ATP as a substrate and these will be described later.

### MantATP Binding to RecG⋅DNA

**Figure 3:**
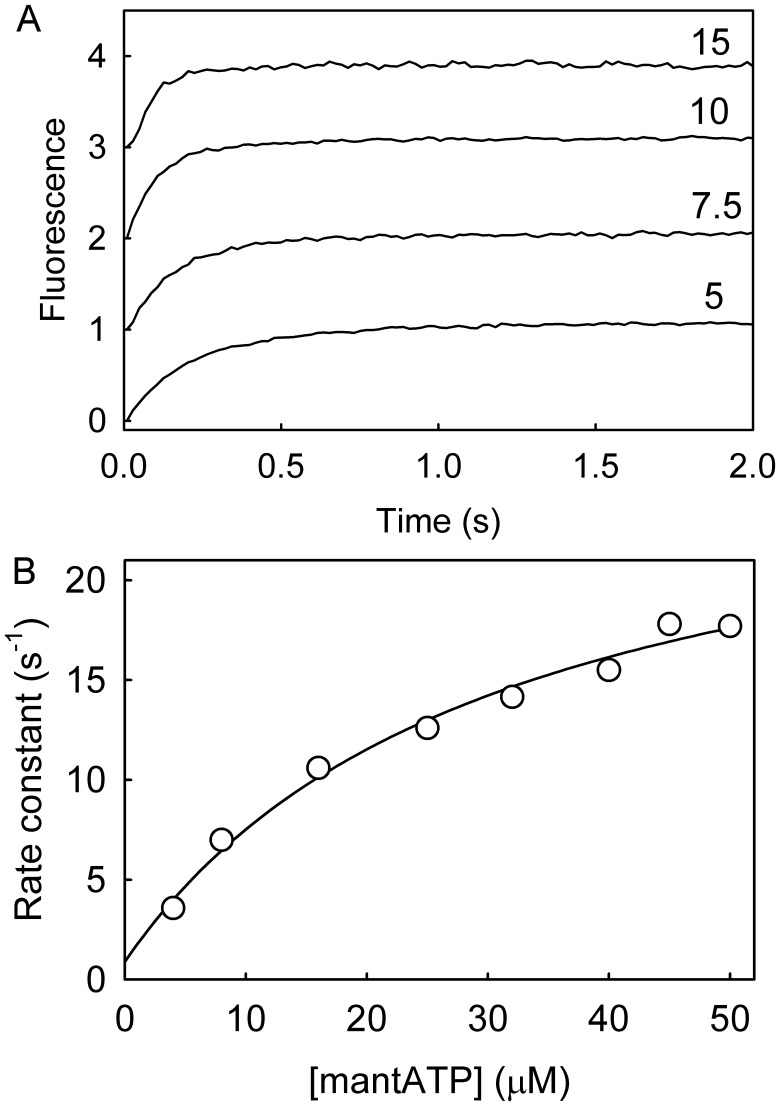
MantATP binding to RecG⋅DNA. MantATP at the micromolar concentrations shown was mixed in the stopped flow apparatus with 0.5 µM RecG and 2.5 µM DNA Junction (A40:B40) at 20°C in the buffer described in materials and methods. Individual traces (offset from each other) were fitted to single exponentials and the dependence of the rate constants on concentration was then fitted by a hyperbola. The points shown are averages of at least 3 measurements. The fit gives 1/*K*_1a_ as 24 (±4) µM and *k*_+1b_ + *k_−_*_1b_ + *k*_+2_ as 26.1 (±1.8) s^−1^ and the intercept was <1 s^–1^ (Scheme in Figure 2B).

MantATP binding to RecG⋅DNA(A40:B40) was measured under pseudo-first-order conditions, that is mantATP in large excess over the protein. Using the stopped-flow apparatus, several concentrations of mantATP were rapidly mixed with RecG⋅DNA and fluorescence followed with time. Each trace was fitted by a single exponential (Figure 3A) and the concentration dependence of the observed rate constants were fit well by a hyperbola (Figure 3B). This suggests that the binding occurs in two steps with the first step being rapid and the second step having the predominant fluorescence change, as shown in the scheme (Figure 2B). As mantATP is also hydrolyzed, the rate of the forward reaction needs to be included, in this case limited by the cleavage step 2, as described later. The fit gives 1/*K*_1a_ as 24 (±4) µM, *k*_+1b_ *+ k*_−1b_ + *k*_+2_ as 26.1 (±1.8) s^−1^ and the intercept gives *k*_−1b_ + *k*_+2_ as <1 s^−1^. Given that *k*_+2_ is 0.2 s^–1^ (see below), *k*_−1b_ is <0.8 s^−1^. Potentially, an alternative explanation for such a hyperbolic relationship could be preferential binding of either the 2′- or 3′-isomer of mantATP and then isomerization of the non-favored isomer. However, the observed rates in these measurements were several orders of magnitude faster than the likely isomerization rate [12]. In order to assess whether the two isomers of mantATP and their interconversion are factors in any fluorescence traces for mantATP, an equivalent measurement was made using 3′-mant-2′-deoxyATP. The data (not shown) was similar to the mixed isomers. Rate constants for individual steps in the mantATPase mechanism are summarized in Table 2.

**Table 2:** Summary of individual rate constants for the hydrolysis cycle of mantATP.

Parameter	Value	S.E.
1/*K*_1a_	24 µM	4 µM
*k*_+1b_	25.1 s^−1^	1.8 s^−1^
*k*_−1b_	<0.8 s^−1^	
*k*_+2_	0.22 s^−1^	0.05 s^−1^
*k*_−2_	<0.01 s^−1^	
*k*_+3_	>10 s^−1^	
*k*_+4_	<1 s^−1^	
*k*_−4_	1.1 µM^−1^s^−1^	0.2 µM^−1^s^−1^

The parameters are defined from the scheme in Figure 2A and B and the values are for 20°C.

**Figure 4:**
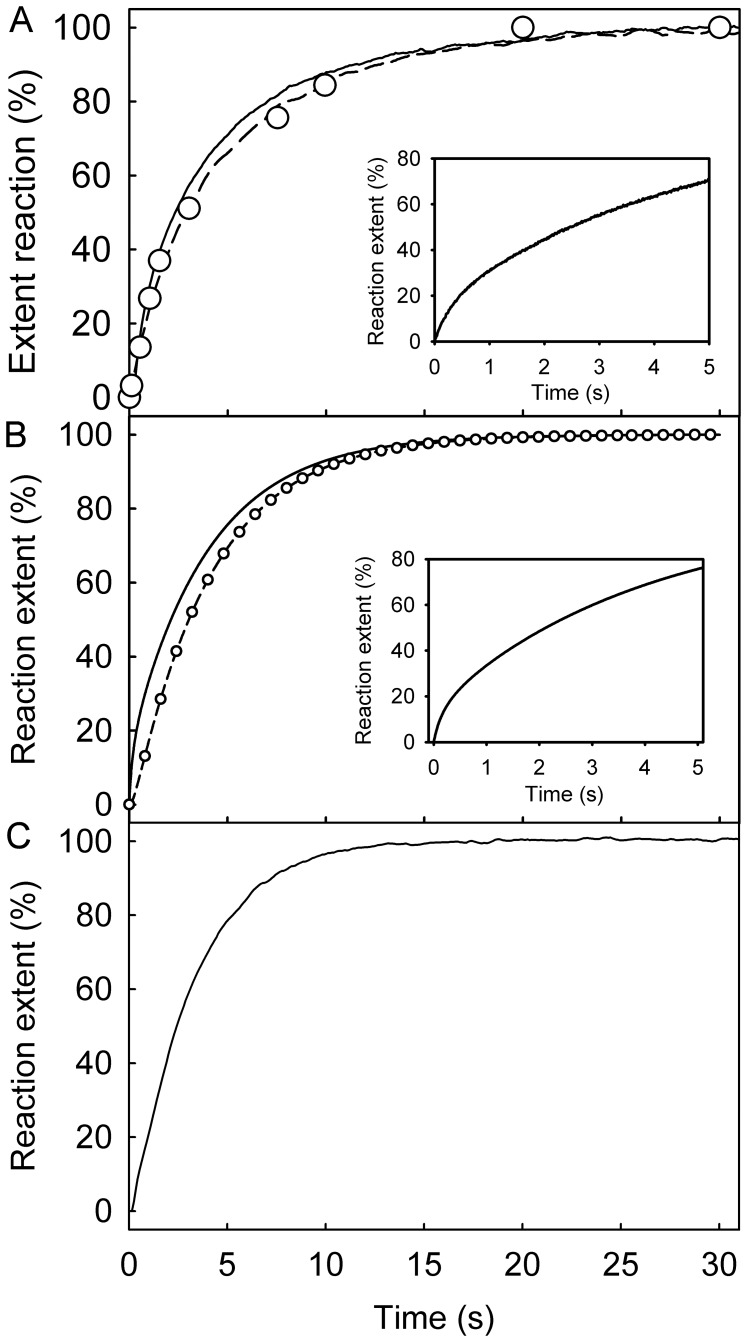
Kinetic measurement of mantATP with excess of RecG and DNA: binding, hydrolysis and P_i_ release. The concentrations for all experiments were 0.5 µM mantATP, 2.5 µM RecG, 5 µM DNA (A40:B40) and 10 µM MDCC-PBP (for P_i_ measurement). All measurements were carried out at 20°C in the buffer described in Materials and Methods. (A) Time course of mant fluorescence (solid line), mantADP formation (circles) and P_i_ release (dashed line), measured as described in materials and methods. The inset shows the initial change in mant fluorescence. (B) Simulation of these time courses, based upon a global model for a single turnover of mantATP as described in the text. (C) The time course of mant fluorescence upon mixing 0.5 µM mantATP and 2.5 µM RecG with 5 µM DNA junction (A40:B40) after a first mixing of 5 µM RecG and 1 µM mantATP and aging for 1 s.

Extra information relating to changes in a single ATPase cycle can be obtained by mixing excess RecG⋅DNA(A40:B40) with mantATP (Figure 4A). The traces showed an initial small increase in fluorescence (inset Figure 4A), which represents ∼20% of the overall change, followed by a slower, but larger increase. The first change in fluorescence is presumably due to binding. After fitting to a double exponential, the observed rate constant for the initial change was linearly dependent on RecG concentration over the small range possible (0.5 µM –2.5 µM), limited by protein precipitation at higher concentrations. Due to the low concentrations, a hyperbolic dependence would not be observed. The observed rate constant of the second increase in fluorescence, 0.22 (±0.05) s^−1^ (Figure 4A), was independent of RecG concentration. This second phase is likely to be due to the cleavage step, leading to the formation of bound mantADP. The fluorescence intensity does not change subsequently because almost all mantADP would remain bound at the concentrations used.

The two phases of this fluorescence increase with excess RecG⋅DNA would therefore relate to formation of bound mantATP (1.5-fold intensity increase relative to free nucleotide) and bound mantADP (4.9-fold). This interpretation was supported by titrations of RecG into the fluorescent nucleotides in the absence of DNA. MantATP exhibited a 1.3-fold increase in fluorescence, while mantADP had a 4.8-fold increase in fluorescence (data not shown).

### Cleavage Step and P_i_ Release

Single turnover measurements of the cleavage step and P_i_ release were performed with excess RecG⋅DNA over mantATP using conditions of the mantATP fluorescence measurements, described above. Quench-flow measurements allowed the formation of nucleoside diphosphate to be monitored with high time resolution. MantATP was rapidly mixed with excess RecG⋅DNA, and the reaction mix was quenched in acid at particular time points. The mix was then analyzed by HPLC to quantify mantATP and mantADP, giving the time course of mantADP formation (Figure 4A). This time course had similar kinetics to the slow phase of the mant fluorescence trace (0.2 s^−1^), supporting the idea that this phase is mantADP formation.

Using the phosphate biosensor, MDCC-PBP [13], the kinetics of P_i_ release were measured under the same conditions (Figure 4A). Fitting the trace by a lag then a single exponential increase gave rate constants of 3.3 s^−1^ and 0.21 s^−1^. This rate constant for the lag was similar to the observed rate constant for binding at this concentration. The exponential increase had the same rate constant for the slow phase of the mant fluorescence and for the quench-flow measurement of mantADP formation. Thus P_i_ release occurs rapidly, following the rate-determining cleavage step.

### MantADP Diphosphate Binding

**Figure 5:**
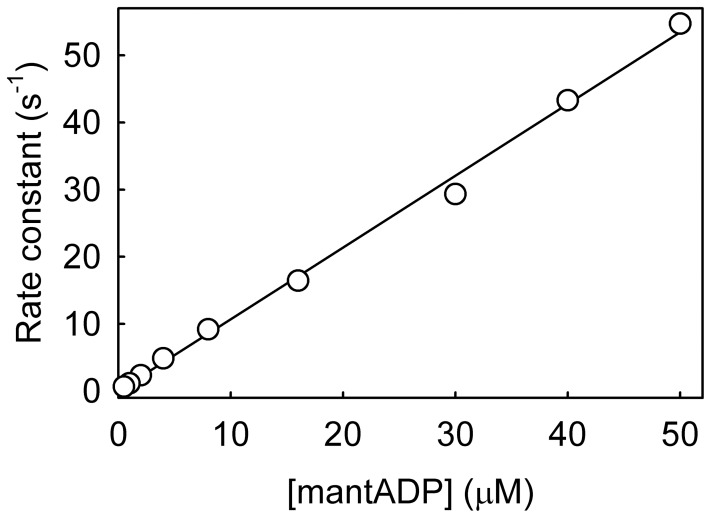
MantADP binding kinetics to RecG⋅DNA. MantADP at various concentrations was mixed in the stopped flow apparatus with 0.5 µM RecG and 2.5 µM DNA (A40:B40) under the conditions of Figure 4. Traces (not shown) were fitted by single exponentials. The rate constants are shown as a function of concentration and the best linear fit, gives a slope of 1.1 (±0.4) µM^−1^s^−1^ and an intercept of <1 s^−1^.

MantADP binding to RecG⋅DNA complex was measured under pseudo-first order conditions by stopped-flow fluorescence. The traces showed a single exponential increase. Figure 5 shows a linear dependence between observed rate constants and mantADP concentration, giving a second order rate constant (*k*_−4_ in the scheme of Figure 2A) of 1.1 (±0.4) µM^−1^ s^−1^ and a dissociation rate constant (*k*_+4_) of <1 s^−1^ from the intercept (Table 2).

Using the same conditions as the hydrolysis and P_i_ release measurements, it was possible to compare the fluorescence intensity seen when mantATP binds. The trace showed a large (4.5-fold) increase in fluorescence, which is consistent with the end point of the fluorescence change of mantATP binding (Figure 4A) being related to bound mantADP.

### Oxygen Exchange Measurements

In order to get information about the reverse of cleavage step 2 (scheme in Figure 2A), phosphate-water oxygen exchange measurements were done using (γ-^18^O_3_)mantATP. Intermediate exchange can occur between terminal oxygens of the nucleotide and water during hydrolysis, giving information about the value of *k*_+3_ */k*_−2_, as previously described [14]. In this case, (γ-^18^O_3_)mantATP was used as substrate and partial hydrolysis occurred in the presence of RecG⋅DNA, as described in the materials and methods section. The distribution are shown in Table S2. The P_i_ had very similar distribution of oxygen-18 as with (γ-^18^O_3_)mantATP, consistent with essentially little reversal of the cleavage step (*k*_+3_ */k*_−2_≥9). This is consistent with a rapid release of P_i_.

### ATPase Measurements with a Three-strand Junction

In the ATPase cycle measurements described above, RecG does not separate any double-stranded DNA (dsDNA) and so it was important to determine whether the kinetic mechanism was similar, when unwinding. Therefore, mantATP binding, quench-flow and phosphate measurements were carried out using with sub-stoichiometric mantATP, but with a non-complementary three-strand DNA junction (A40:B40:C19) to form a lagging strand duplex (as defined in Figure 1A). With excess mantATP, the duplex arm of this junction was unwound by the helicase, leaving RecG bound to the two-strand junction with the third strand displaced. With sub-stoichiometric mantATP, there was only a single turnover, so only partial unwinding of this DNA substrate occurred. The kinetics (Figure S2) were similar to those with the two-strand junction with mantATP binding at 4.2 (±0.4) s^−1^, hydrolysis at 0.39 (±0.1) s^−1^ and fast P_i_ release. Thus, the DNA substrate does not affect the ATPase kinetics significantly.

### A Model of the ATPase Cycle

In order to test the data obtained with mantATP for consistency, a simple kinetic model for the ATPase mechanism was used to simulate the data in Figure 4A. The model was based on the scheme in Figure 2A, but included a two-step triphosphate binding as in Figure 2B. The model assumed that P_i_ release is irreversible and mantADP remains bound under these conditions as its *K*_d_ (<50 nM) is much less than the experimental concentrations. The difference in fluorescence intensity between protein-bound mantATP and mantADP was included in the modeling with bound mantATP representing 20% of the overall increase, as shown experimentally. The best-fit simulation is shown in Figure 4B, which gave mantATP binding at 5.7 s^−1^, followed by a rapid step (“conformation change”) of 30 s^−1^, hydrolysis at 0.25 s^−1^ and P_i_ release is fast (>10 s^−1^). This shows that the data fit to a model with rate-limiting hydrolysis. There is a discrepancy with the fit to the data at long times, as the experimental traces are slightly biphasic, which could be due to a small proportion of the protein having modified activity due to damage, for example.

### Measurements in the Absence of DNA

The kinetics of specific steps of the ATPase cycle were investigated in the absence of DNA, to determine which are modulated by the interaction with DNA. For the association measurements between RecG and mantATP, RecG was mixed with excess mantATP in the stopped-flow apparatus and the fluorescence followed with time. Following fitting by single exponentials, there was a linear relationship between the observed rate constant and nucleotide concentration (Figure S3A). Interpreting the increase in terms of single-step binding gives a second order association rate constant of 0.4 (±0.15) µM^−1^s^−1^ and an intercept of 1.7 (±0.6) s^−1^ and, therefore, a *K*_d_ of 4.25 µM, not greatly different from the values with DNA.

As done for the RecG⋅DNA complex, measurements were also taken with an excess of RecG over mantATP (Figure S3B). A large, relative rapid, increase in mant fluorescence was observed followed by a small gradual increase. The rate constants are consistent with those at excess mantATP. Under the same conditions of excess RecG over mantATP, mantATP cleavage (acid quench) and P_i_ release were measured (Figure S3B). The hydrolysis time course, measured by manual quench, was similar to that obtained for P_i_ release, although the reaction did not go to completion in the time scale of the measurements (3000 s). The cleavage step rate constant was 4×10^−4^ s^−1^, ∼10^3^ slower than in the presence of DNA. The phosphate measurement suggested that P_i_ release follows rapidly after cleavage. The cleavage step was greatly stimulated by DNA.

As the cleavage step is so slow in the absence of DNA, a double-mix, stopped-flow experiment was performed in order to see if the biphasic increase in mant fluorescence could be separated into the individual processes of mantATP binding and hydrolysis. An excess of RecG was mixed with mantATP and this mix aged for 1 s to allow binding but not hydrolysis. This solution was then mixed with DNA. It was demonstrated in a separate measurement that DNA binding was fast for a variety of junctions (Table S3). The fluorescence trace (Figure 4C) showed a single exponential increase in mant fluorescence with a rate constant of 0.3 (±0.04) s^−1^. This rate was similar to that of the slower phase, measured above, consistent with this phase being due to DNA-induced hydrolysis.

### Measurements with Unlabeled ATP and ADP

There is no intrinsic change in protein tryptophan fluorescence upon binding ATP or ADP. Furthermore, the weak affinity for ATP means it was not feasible to form the RecG⋅DNA⋅ATP complex quantitatively in order to measure the steps in the ATPase cycle, as could be done with mantATP. However, it was possible to measure two key processes, P_i_ and ADP release and so indirectly obtain information about the other steps. These are important measurements, given the different affinities between labeled and unlabeled nucleotides. Through these measurements tthe mechanism described with mantATP can be related to that with ATP.

**Figure 6:**
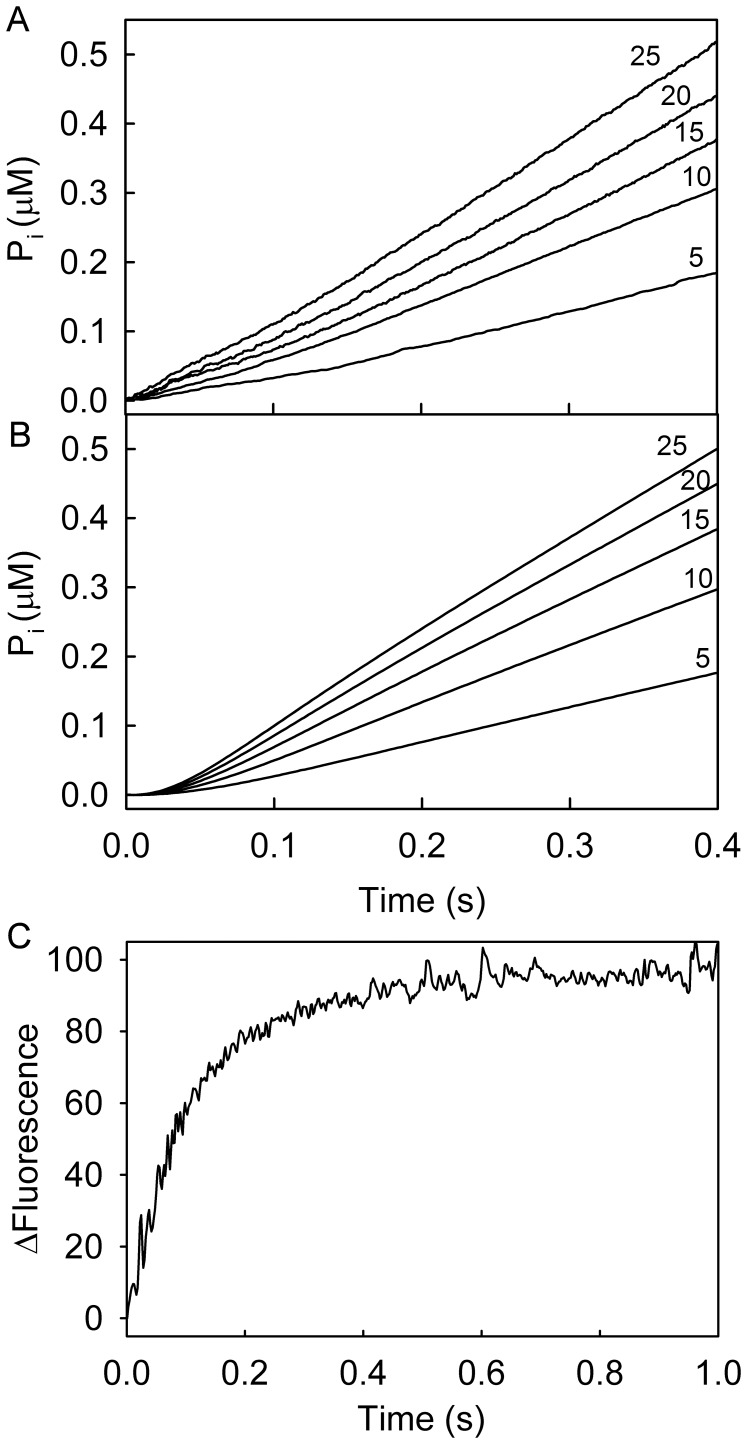
ATPase cycle measurements with unmodified nucleotides. (A) Phosphate measurements at different ATP concentrations. 0.5 µM RecG, premixed with 2.5 µM DNA (A40:B40), was then mixed in the stopped-flow apparatus with ATP at the micromolar concentrations shown, in the presence of 10 µM MDCC-PBP. (B) Best fit time courses of the P_i_ release, based upon a model of the ATPase cycle, as described in the text. Curves were obtained by glogal fitting the curves at different concentrations, using Global kinetic Explorer [40]. Note that in comparison, the experimental time courses show a small transient of P_i_ at times <100 ms. This is likely to be due to free P_i_ and this behavior, overlaying a lag, has been observed previously in multi-turnover measurements [13]. (C) ADP Release kinetics. 10 µM RecG⋅DNA (A40∶B40) was pre-bound with 10 µM ADP before mixing with an excess of mantADP (20 µM): these concentrations are final in the mixing chamber. The increase in fluorescence was fitted to single exponential giving the dissociation rate constant of 11.4 (±2.2) s^−1^. Rate constants were independent of mantADP concentration in the range of 20–80 µM.

The rate of P_i_ release was measured after mixing RecG⋅DNA with excess ATP, using the phosphate biosensor, in the stopped-flow apparatus (Figure 6A). The traces showed a slight lag, followed by a fairly linear rate of P_i_ release. Both phases are dependent on ATP concentration. The lag decreased, while the rate of the linear phase increased with greater concentrations of ATP. The lag is due to the steps prior to P_i_ release, namely ATP binding and cleavage. These traces represented multiple ATP turnovers and showed no burst of P_i_ release during the first turnover: therefore ADP release is not rate limiting in the cycle. Such a burst phase would result from the relatively fast P_i_ release of the initial cycle before the P_i_ release of subsequent cycles was limited by slow ADP release.

A scheme of the ATPase cycle (Figure 2) was used to simulate the data to assess the individual rate constants. The data do not distinguish between cleavage and P_i_ release steps, so the simulation assumed a slower hydrolysis step is followed by rapid P_i_ release, as determined by measuring these steps with the mantATP. The simulated traces (Figure 6B) were obtained from a global fit to the experimental data. This was done by allowing only the rate constants that have maximum influence on the curve shapes to vary. Cleavage and P_i_ release were assumed to be irreversible. For the fit, P_i_ release was fixed at 100 s^–1^, ADP release at 12.5 s^–1^, as the fitting was relatively insensitive to changes in these constants. ADP binding was therefore fixed at 1.7 µM^−1^s^–1^ to be consistent with the value of the *K*_i_ determined for ADP (Table 1b). The resulting best fit gave rate constants for ATP binding as 0.65 (±0.02) µM^−1^s^−1^, dissociation of ATP as 10.0 (±0.6) s^−1^, cleavage as 7.1 (±0.1) s^−1^. The fit gave a cleavage rate constant similar to the value of *k*_cat_ from the steady-state analysis (Table 1a). The rate constant for ADP release may be only slightly larger than that of cleavage step.

The equivalent oxygen exchange experiment was performed with ATP hydrolysis, as described above with mantATP, and shown in Table S2. There was also a low extent of exchange supporting the idea of rapid P_i_ release, assumed above.

### ADP Dissociation Kinetics

A direct measurement of ADP dissociation kinetics was possible because of its relatively tight binding to RecG, which allowed quantitative formation of the ADP complex. RecG⋅DNA was pre-bound to ADP before mixing with a large excess of mantADP in the stopped-flow apparatus. The very tight binding of mantADP (Table 1b) made that a suitable trap for free RecG⋅DNA, after dissociation of ADP. The rate constant for dissociation was determined from a single exponential fit as 11.4 (±2) s^−1^ (Figure 6C). This value fits well with the model whereby the dissociation rate constant is calculated as ≥12.5 s^−1^. Using the dissociation rate constant (11.4 s^−1^) and the *K*_i_ value (7.3 µM), the estimated association rate constant is 1.6 µM^−1^s^−1^.

Overall, the data presented here with unlabeled nucleotides fits to the model described by the mant nucleotides, whereby there is rate limiting cleavage. By measuring the product release (P_i_ and ADP) with the unlabeled nucleotides, it is clear that ADP release is fast and that cleavage is the limiting step.

### Unwinding of Homologous DNA Junctions

**Figure 7:**
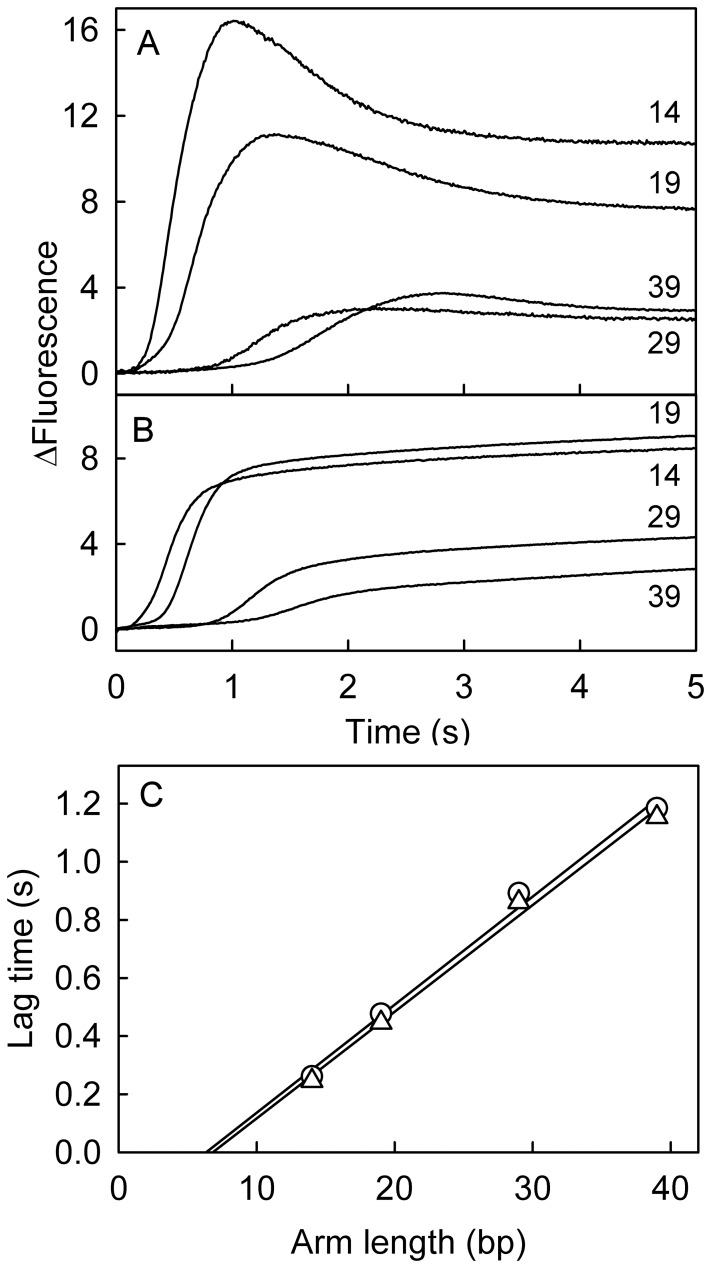
Unwinding 4-strand complementary junctions. (A) Fluorescence changes during unwinding under single turnover conditions junctions (A:B′:C:D) of varying arm lengths (shown in b.p.), labeled with Cy3 and Dabcyl on the template strand duplex. Final concentrations are 30 nM DNA junction, 60 nM RecG and 200 µM ATP. The reaction was initiated by rapid mixing with ATP. (B) Fluorescence changes during unwinding under single turnover conditions of 4-strand complementary junctions (A∶B′∶C∶D), of varying arm lengths (shown in b.p.), labeled with Cy3 and Dabcyl on the leading strand duplex. Final concentrations DNA junction 30 nM, RecG 60 nM and ATP 200 µM. The reaction was initiated by rapid mixing with ATP. Fluorescence levels are expressed relative to the starting value. (C) Change in lag durations with arm length of complementary junctions.: 4-strand with lagging strand labeled (triangles), 3-strand with lagging strand labeled (circles) and 4-strand with leading strand labeled (squares). The length of lags were obtained by the intercept of best fits to the lag phase and the tangent to the rise phase, Lines are linear fits to the data points, and the reciprocal gives rates of unwinding 26.9 (±1.6) bp s^−1^ for the lagging strand and 26.9 (±1.6) bp s^−1^ for the leading strand.

After measuring the ATPase kinetics and determining how they relate to interactions with DNA, it is important to correlate helicase activity with detailed enzymatic activity. Previously, unwinding measurements were reported with non-complementary three-way junctions [10]. RecG activity requires the substrate DNA to be a three-way junction, but the junction arms can be made up of completely duplex DNA or one arm, or both arms can be single strand, as used above. This diversity of DNA structures mimics the potential DNA substrates in the cell, depending on the precise extent of strand replication prior to the premature termination. It was therefore of interest to measure RecG activity on four-strand homologous junctions (Figure 7). The final products of unwinding are fully complementary, double strands. The kinetics of unwinding such DNA were measured with a Cy3/Dabcyl fluorophore/quencher pair at the distal end of the lagging strand, as done previously [10], [15]. In such substrates, the Dabcyl interacts with the Cy3 and quenches its fluorescence. However, on completion of unwinding, the two labels separate and the fluorescence increases (Figure 1B).

Rapid mixing of preformed complexes, RecG⋅DNA junctions (A∶B′∶C∶D, Figure 1A), under conditions of single turnover with respect to DNA, and excess ATP resulted in fluorescence time courses showing a lag phase, followed by an increase in fluorescence (Figure 7A). After a transient peak in fluorescence, the intensity dropped to a constant lower level. The magnitude of the overall fluorescence change decreased as the length of the substrate junction arms increased. The duration of the lag phase increased linearly with duplex length (Figure 7C), giving a translocation rate of 26.9 (±1.6) bp s^−1^. This rate is similar to that previously reported for non-complementary junctions [10].

The measurement was repeated with the two labels at the distal end of the leading strand duplex (Figure 7B) to determine if unwinding of both arm duplexes is completely synchronous. An ATP-dependent increase in fluorescence following a lag period was observed, similar to when the lagging strand was labeled. The amplitudes and shapes of the traces were considerably different in this case as there was a slow increase in fluorescence after the main rise phase. This presumably is due to different interactions between Cy3 and RecG, depending on the arm labeled, which imposes a different fluorescence intensity change. Analyzing the data as before, a plot of lag durations against substrate junction arm length showed a linear relationship (Figure 7C), giving a translocation rate of 27.2 (±1.5) bp s^−1^. Thus, unwinding the lagging and leading arm duplexes occurs at a similar rate, consistent with the processes being fully synchronized and controlled by the pulling of the template strand across the RecG surface in response to changes in the ATP binding site.

### ATP Hydrolysi0073 Rate during Unwinding

**Figure 8:**
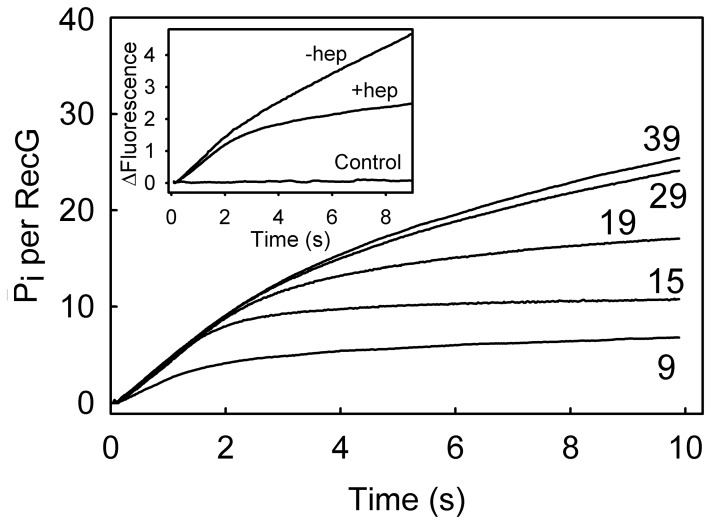
ATP hydrolysis during unwinding 4-strand complementary junctions. Junctions of varying arm lengths (shown in b.p.) were used to measure the rate of ATP hydrolysis during unwinding. RecG was pre-incubated with junction and the reactions were initiated by rapid mixing with ATP and heparin. Final concentrations are 200 nM DNA junction (A∶B′∶C∶D), 10 nM RecG, 200 µM ATP, 1 mg ml^−1^ (55.5 µM) heparin and 5 µM MDCC-PBP. The reactions were performed in the presence of a P_i_ mop, described in materials and methods. Inset: Effect of heparin on ATPase activity of RecG during unwinding. The reactions were initiated by rapidly mixing the pre-incubated RecG.DNA (A40:B′40:C19) with ATP with or without heparin. In another measurement (“control”), RecG was pre-incubated with heparin and then rapidly mixed with DNA junction (A40:B′40:C19) plus ATP. Final concentrations are 200 nM DNA junction, 10 nM RecG, 200 µM ATP, 2 mg ml^−1^ (111 µM) heparin and 5 µM MDCC-PBP with P_i_ mop.

The rate of ATP hydrolysis and total ATP usage during unwinding were measured using the phosphate biosensor (Figure 8), following rapid mixing of ATP with the RecG⋅DNA complex. A measurement of P_i_ production during unwinding showed a break point in the trace when heparin was used as a trap (Figure 8 inset). Heparin is a potent inhibitor of many helicases, mimicking the DNA substrate and binding tightly to the enzyme, once the latter dissociates from the DNA, and so prevents rebinding of the helicase to fresh DNA substrates. The initial, rapid phase, which represents unwinding of the first DNA junction, was unaffected by the presence of heparin. However, the subsequent change in rate is slow, suggesting that the interaction with heparin is slow, possibly due to slow release of RecG from the end of the DNA, or due to inherently slow interaction of RecG with heparin. RecG will continue to hydrolyze ATP while bound to the DNA substrate. Therefore, this does not produce an abrupt change in rate at the end of unwinding the first DNA substrate.

The ATPase measurement was repeated with different lengths of lagging and leading strands, as used in the unwinding measurements, in the presence of heparin (Figure 8 main panel). The break point increases with length, albeit not sharply. The initial rate was independent to the length of the substrate junction arms, except the shortest, suggesting a constant rate of ATP hydrolysis during unwinding. The 9-bp arm may be too short to have full interaction with RecG. The rate of ATP hydrolysis during this unwinding phase was 5.8 s^−1^. Taking the mean unwinding rate of 27 bp s^−1^, gives a coupling ratio around 4 bp ATP^−1^ for the fully complementary junctions. The ATPase rate measured here is similar to the steady-state and hydrolysis rate constants measured in the above sections, supporting the idea that the ATP hydrolysis reaction is not greatly affected by the type of junction.

## Discussion

Many helicases have significant unwinding activity only when associated as part of a larger complex, either as a homo-oligomer or with additional proteins. For example, PcrA on its own is a poor helicase [16], [17] but, when associated with an plasmid replication initiator protein such as RepD, its activity is greatly increased and it has a processivity up to plasmid lengths of DNA [16]. This is not the case with RecG, where the helicase alone can readily catalyze DNA unwinding and it must be able to remain bound to the DNA junction on its own otherwise the replication fork could collapse. This does not preclude the helicase interacting with other proteins, but this is more likely to occur during recruitment. Such a behavior requires the helicase to bind to the DNA rapidly and stably. Low and high affinity states allowing translocation may then be linked to stages of the ATPase cycle. To explore these possibilities further, the individual steps in the ATPase cycle were measured, along with the helicase activity on DNA substrates, which mimic those in the cell.

Four-strand oligonucleotide junctions (Figure 1A) were used to measure unwinding kinetics, as these structures more closely mimic the natural substrates than our previous study of RecG [10]. By differential labeling of lagging and leading strands, it was shown that both arms are unwound synchronously. This would be expected for a mechanism by which the parental duplex (template arm) is translocated, drawing the two daughter strands towards the helicase. The motor domain translocates dsDNA and draws the substrate across the wedge domain, which catalyzes the rearrangement of the substrate and so translocates the Holliday-junction-like structure. The motor domain is likely to be largely unperturbed by the DNA structure that interacts with its accessory domain, as it only binds to the parent strand.

When unwinding four-strand homologous junctions a mean unwinding rate of 27 bp s^−1^ and an ATP turnover rate of 5.8 s^−1^, indicated a coupling ratio of ∼4 bp per ATP hydrolyzed. This is somewhat higher than the value of ∼3 reported previously with a three-strand non-complementary substrate [10]. In any case, the data suggest that for a variety of DNA junctions the coupling ratio is significantly greater than 1, the value found with several helicases, particularly from Superfamily 1. Thus, PcrA has a ratio of 1 for both single and double stranded DNA translocation [18], [19], as suggested from structural studies, which show individual base binding pockets [20]. The complete interactions of RecG motor domain with DNA are not clear from the structure [8], although the DNA is double-stranded in this region, therefore the motor domain is unlikely to have specific interactions with bases. The main factor limiting the step size may well be the size of the conformation change as the motor domain reaches along the dsDNA. These changes in structure may involve specific interactions with DNA grooves and DNA sliding. The binding and release, or sliding, would be linked to stages in the ATPase cycle. With the 4-way homologous junction there is no net formation of ssDNA and no net base pair separation, so that the energetics of the translocation are not significant, in relation to the energy available from hydrolysis of an ATP molecule.

The detailed ATPase mechanism was investigated by measuring individual rate constants to determine which steps control the rate of overall reaction and which were modulated by DNA, and, therefore, which nucleotide intermediates are the main ones present during translocation. These data are summarized in Table 2 and can be used to determine when and where conformation changes may occur. Mant-nucleotides were used for this together with a two-strand junction, so there were fluorescent signals for different steps of the cycle to establish a full kinetic mechanism for the ATPase cycle. Measurements with the natural unlabeled nucleotides were also done, when possible, to address the differences in rate constants due to ribose-linked labels and test the ATPase model from derived from the mant measurements.

MantATP has a five-fold higher affinity and 30-fold lower ATPase activity than ATP. The low activity of mantATP is due both to slower cleavage and mantADP release. However, both these key rate constants can be measured for the natural substrate. Importantly, the increased mant affinities allow quantitative complex formation with RecG and therefore all parts of the ATPase cycle could be investigated with this nucleotide: this would not be possible with unmodified nucleotides. This allowed the complete the ATPase cycle to be modeled and then tested against all available data with the unlabeled ATP. While absolute rate constants may differ, the key components of the motor, such as rate limiting step and DNA activated steps are not necessarily affected by these affinity changes. Significant changes in ATPase activity and nucleotide affinity have been reported with other motor proteins [21] and helicases: an RNA helicase showed a two-fold changes in affinity and activity [22]. In the case of PcrA and mantADP, an increase in affinity of over an order of magnitude was observed [23].

Using the mantATP it was possible to measure binding, hydrolysis and P_i_ release under the same conditions and show that hydrolysis is rate limiting followed by very rapid P_i_ release. These key features were then derived with unlabeled ATP. In order to achieve this, the kinetics of P_i_ release were measured at various concentrations of ATP. The traces were fitted very well using the model derived from mantATP measurements, namely rate limiting hydrolysis, followed by rapid P_i_ release with a relatively tight ADP affinity. Furthermore, the absence of a burst phase in the P_i_ release data confirms that ADP release does not contribute significantly to rate limitation. Even so, ADP release is not rapid suggesting that there could be more complexity in the release of this product. The kinetic measurements suggest that RecG⋅DNA⋅ATP is the major intermediate present during translocation and unwinding. There is also be significant RecG⋅DNA⋅ADP and so these two intermediates are likely to represent the main conformations, whose interconversion produces the movement along DNA. Rate limitation by hydrolysis was also reported for PcrA [23] and an RNA helicase, DbpA [22]. The modeled nucleotide association rate constants are well below those expected for a diffusion-controlled process, a situation observed with various other helicases [22], [23], [24], [25]. In any case, at physiological concentrations of millimolar ATP, its binding would still be rapid, relative to cleavage.

DNA accelerates the ATPase rate by enhancing the rate of the cleavage step. Due to the limited structural information, it is not directly clear what DNA-driven changes occur. It was proposed that the DNA binding site is directly linked with the ATP catalytic site [9] providing a likely mode for this rate enhancement. Additionally, the ATPase rate maybe regulated by controlling access of Mg^2+^ to the catalytic site, as proposed for PcrA [26]. An equivalent residue (K402) exists in RecG to the one implicated in PcrA.

The relatively tight binding of ADP would lead to the ATP-bound and ADP-bound states being the main states with similar concentrations at steady state. It is likely that the main structural differences that are responsible for translocation are shown in these two states. Therefore, it is likely that ADP release correlates with one of the significant structural changes. This is supported by measurements of the mant fluorescence, which showed a large fluorescence change following binding and presumably representing a conformation change during hydrolysis. Such a conformation change is likely to be reversed upon ADP release, so the main structural changes occur upon hydrolysis and ADP release. However, an additional structural change upon nucleotide binding cannot be discounted with our data, given the two steps required by the data (Figure 3). This model is different to known SF1 helicases (PcrA, Rep and UvrD) [27], [28], [29], whereby binding and hydrolysis are likely to be the significant structural changes. The larger translocation step size (4 bp ATP^−1^) of RecG will likely result from this different mechanism compared to SF1 helicases with a typical step size of 1 bp ATP^−1^ [19], [30]. The ATPase cycle of a DEAD-box superfamily 2 RNA helicase, DbpA, has also been studied [22], [31]. This helicase is likely to have a two-step ADP release similar to that proposed for RecG but both hydrolysis and phosphate release may contribute to rate limitation as they occur at similar rates. There is also significant reversal of the hydrolysis step. While there are few examples of investigating the individual steps of a helicase ATP cycle, many studies have measured the overall ATPase rate. In all cases this is enhanced by the presence of the appropriate nucleic acid substrate.

Further high resolution studies looking at the conformation changes during the translocation mechanism is required to determine an accurate mechanism like those for SF1 enzymes. This highlights the different ways in which DNA-based motor proteins function, even when catalyzing similar processes.

## Materials and Methods

### Materials

RecG from *T. maritima* was expressed and purified as described previously [10] with the modification that the final blue Sepharose column was not required as the protein was >95% pure following the first two columns. Coumarin-labeled phosphate binding protein (MDCC-PBP) was prepared as described [32], [33]. (^18^O_4_)P_i_ and (γ-^18^O_3_)ATP were synthesized from (^18^O)water (97% enriched) as described [34]. MantATP, mantADP and (γ-^18^O_3_) mantATP were synthesized from their parent nucleotides by a modification of the method of Hiratsuka [11], [35], [36]. Labeled and unlabeled oligonucleotides were from Eurogentec Ltd (Southampton, UK). Oligonucleotide junctions with sequences defined in Table S1 were prepared by assembling separately equimolar amounts of the oligonucleotides corresponding to the template and leading strand arms of the junctions. RecG was mixed with the substrate complexes and stored on ice prior to use. Oligonucleotide junctions were formed just prior to use by pre-incubating the DNA in the reaction buffer at 20°C for 10 min. All oligonucleotides, labeled with the fluorophore Cy3, were modified at the 5′-position and all oligonucleotides, labeled with the quencher Dabcyl, were modified at the 3′-position. All other biochemical reagents were from Sigma.

### Oxygen Exchange Measurements

For intermediate exchange during ATP hydrolysis, 0.5 µM RecG and 1 mM (γ-^18^O_3_)ATP or (γ-^18^O_3_)mantATP were incubated for 10 min (+1 µM DNA A40∶B40, Table S1) or 20 min (- DNA) at 25°C in a volume of 100 µl in the buffer described below. P_i_ was analyzed for distribution of different ^18^O-labeled species on a mass spectrometer as described previously [14], [37] except that during purification, the ^18^O-labeled P_i_ was detected by MDCC-PBP, rather than radioactive tracers.

### Quenched Flow Measurements

These were carried out using a HiTech RQF-63 apparatus using different length loops and flow rates to age reactions before quenching using 10% perchloric acid. Samples were analyzed for the ratio of mantATP to mantADP by HPLC, as described [23].

### Optical Measurements

Stopped-flow experiments were performed in a HiTech SF61DX2 apparatus (TgK Scientific Ltd, Bradford-on-Avon, UK) with a mercury-xenon light source and HiTech KinetAsyst 3 or Kinetic Studio 1 software. For MDCC-PBP fluorescence, the excitation wavelength used was 436 nm and a 455 nm cut-off filter (Schott glass) used to collect emitted light. The signal was calibrated using known concentrations of P_i_. The solutions contained the P_i_ mop, to minimize phosphate contamination and comprising 0.01 unit ml^−1^ bacterial purine nucleoside phosphorylase and 200 µM 7-methylguanosine [38]. For mant fluorescence, 366 nm was used to excite and a 400 nm cut-off filter (Schott glass) used to collect light. Measurements of Cy3 fluorescence used 547 nm excitation and a 570 nm cut-off filter. In experiments described, the quoted concentrations are those in the mixing chamber, except where stated. Data were fitted to theoretical curves using the HiTech software or Grafit.

Steady-state fluorescence was measured using a Cary Eclipse fluorimeter (Varian) with a xenon light source. Absorbance spectroscopy was performed using a Beckman DU640 spectrophotometer.

### Kinetic Measurements

All reactions with RecG and DNA were done at 20°C in a buffer containing 50 mM Tris⋅acetate (pH 8.0), 30 mM potassium acetate, 3 mM magnesium acetate and 1 mM DTT. ATPase measurements using MDCC-PBP were taken in the presence of a P_i_ mop, as described above. Steady-state ATPase measurements were taken in a solution (60 µl) at 20°C, containing 10 nM RecG, 500 nM DNA junction (A40∶B40, see Table S1) and 10 µM MDCC-PBP with varying nucleotide concentrations as substrate or inhibitor [23].

### Analysis of Kinetic Data

Data were fitted to theoretical equations using the stopped-flow software or Grafit [39]. Kinetic simulations were performed using Berkeley Madonna (Version 8.3, University of California at Berkeley) and using Global Kinetic Explorer (Kintek) [40]. Global fits were performed using Global Kinetic Explorer.

## Supporting Information

Figure S1 **Steady state ATPase activity of RecG.** The measurements were carried out at 20°C with solution conditions as described in Materials and Methods with 10 nM RecG, 500 nM DNA Junction (A40:B40), 10 µM MDCC-PBP and triphosphate nucleotide at the concentrations shown. (A) Steady-state measurements for ATP. The lines are best fits to the Michaelis-Menten equation and give a *K*_m_ of 9 (±2) µM and a *k*_cat_ of 8.3 (±1.7) s^−1^. (B) Steady-state measurements for mantATP. The best fit gives a *K*_m_ of 1.9 (±0.6) µM and a *k*_cat_ of 0.3 (±0.1) s^−1^.
(TIF)

Figure S2 **Kinetic measurements of mantATP with excess of RecG and a Three-strand DNA Junction.** The concentrations for all experiments were 0.5 µM mantATP, 2.5 µM RecG, 5 µM DNA Junction (A40:B40:C19) and 10 µM MDCC-PBP (for P_i_ measurement). All measurements were carried out under the conditions of Figure 4. Time course of mant fluorescence (continuous line), mantADP formation (circles) and P_i_ release (dashed line). The insert shows the initial change in mant fluorescence.
(TIF)

Figure S3 **Kinetic measurements in the absence of DNA.** (A) Association kinetics with mantATP. Dependence of the observed rate constants on mantATP concentration from mixing 0.5 µM RecG with excess mantATP under the same conditions as Figure 3. Points shown are typically an average of three individual measurements. The best linear fit gives a slope of 0.4 (±0.15) µM^−1^ s^−1^ and intercept 1.7 (±0.6) s^−1^. (B) Fluorescence trace upon mixing 2.5 µM RecG with 0.5 µM mantATP under the conditions of Figure 4. The trace was fitted by an exponential and a slope giving rates of 0.42 (±0.12) s^−1^ for the former. Note that as the fluorescence slowly increases beyond the time course, it was not possible to calibrate the ordinate. Inset: Hydrolysis and P_i_ release for these conditions. The circles represent single time points for a quenched-flow measurement of mantADP formation. P_i_ release kinetics (continuous line) were measured for the same mixture containing 10 µM MDCC-PBP.
(TIF)

Table S1 **Oligonucleotide sequences**. They are all are written 5′ to 3′. The E19 oligonucleotide is complementary to the B40 oligonucleotide and was used to create 4 strand non-complementary junctions.
(PDF)

Table S2 **Oxygen exchange during ATP or mantATP hydrolysis by RecG: distributions of oxygen-18 in the P_i_ product.** Experiments were performed as described in Materials and Methods. The data were corrected for isotopic enrichment of the starting nucleotide, 93% for (γ-^18^O_3_)mantATP and 98% for (γ-^18^O_3_) ATP. The table shows the distributions of isotope in the product P_i_ after unlabeled P_i_ was subtracted (along with natural abundance in the (^18^O_1_)P_i_ position), likely to be mainly contamination. Each distribution is the average of three mass spectral assays. Thus the distributions are for the product P_i_ as though the starting enrichment was 100%. In all cases there was very little oxygen exchange as shown by the large peak of (^18^O_3_)P_i_, in which all three γ-oxygens of ATP are retained. The distributions were then used to compute the ratio of rate constants for P_i_ release (*k*_+3_) and on-enzyme ATP resynthesis (*k*_−2_) as in Figure 2 [14], [37]. The P_i_ from ATP hydrolysis consistently gave an abnormal distribution (as shown by higher percentage of (^18^O_1_)P_i_, suggesting the possibility of a second minor activity. However, the analysis assumed a single pathway.
(PDF)

Table S3 **Kinetic parameters describing RecG binding to various model substrates under pseudo-first order conditions.** The junctions are made up from the individual oligonucleotides, as defined in Table S1, but with a Cy3 at the 5′-end of the single strand part of the junction. Binding kinetics were measured with conditions as described in the Materials and Methods at 10 nM RcG and varying the junction concentration in the range 100 nM to 1000 nM. After fitting the fluorescence curves to single exponentials, the observed rate constants were plotted as a function of DNA concentration. Assuming a single step binding, the rate constants were obtained and their ratio gave the *K*_d_ values. Some experiments were done in the presence of 200 µM adenosine nucleotide. The major differences are in the association rate constant: the dissociation varies little.
(PDF)

## References

[1] Cordin O, Banroques J, Tanner NK, Linder P (2006). The DEAD-box protein family of RNA helicases.. Gene.

[2] Bennett RJ, Keck JL (2004). Structure and function of RecQ DNA helicases.. Critical Reviews in Biochemistry and Molecular Biology.

[3] Flaus A, Martin DM, Barton GJ, Owen-Hughes T (2006). Identification of multiple distinct Snf2 subfamilies with conserved structural motifs.. Nucleic Acids Research.

[4] Flaus A, Owen-Hughes T (2004). Mechanisms for ATP-dependent chromatin remodelling: farewell to the tuna-can octamer?. Curr Opin Genet Dev.

[5] Cox MM, Goodman MF, Kreuzer KN, Sherratt DJ, Sandler SJ (2000). The importance of repairing stalled replication forks.. Nature.

[6] McGlynn P, Lloyd RG, Marians KJ (2001). Formation of Holliday junctions by regression of nascent DNA in intermediates containing stalled replication forks: recG stimulates regression even when the DNA is negatively supercoiled.. Proceedings of the National Academy of Science USA.

[7] McGlynn P, Mahdi AA, Lloyd RG (2000). Characterisation of the catalytically active form of RecG helicase.. Nucleic Acids Research.

[8] Singleton MR, Scaife S, Wigley DB (2001). Structural analysis of DNA replication fork reversal by RecG.. Cell.

[9] Mahdi AA, Briggs GS, Sharples GJ, Wen Q, Lloyd RG (2003). A model for dsDNA translocation revealed by a structural motif common to RecG and Mfd proteins.. Embo J.

[10] Martinez-Senac MM, Webb MR (2005). Mechanism of translocation and kinetics of DNA unwinding by the helicase RecG.. Biochemistry.

[11] Hiratsuka T (1983). New ribose-modified fluorescent analogs of adenine and guanine nucleotides available as substrates for various enzymes.. Biochimica Biophysica Acta.

[12] Eccleston JF, Moore KJM, Brownbridge GG, Webb MR, Lowe PN (1991). Fluorescence approaches to the study of the p21ras GTPase mechanism.. Biochemical Society Transactions.

[13] Brune M, Hunter JL, Corrie JET, Webb MR (1994). Direct, real-time measurement of rapid inorganic phosphate release using a novel fluorescent probe and its application to actomyosin subfragment 1 ATPase.. Biochemistry.

[14] Phillips RA, Hunter JL, Eccleston JF, Webb MR (2003). The mechanism of Ras GTPase activation by neurofibromin.. Biochemistry.

[15] Toseland CP, Webb MR (2010). Fluorescence tools to measure helicase activity in real time.. Methods.

[16] Soultanas P, Dillingham MS, Papadopoulos F, Phillips SE, Thomas CD (1999). Plasmid replication initiator protein RepD increases the processivity of PcrA DNA helicase.. Nucleic Acids Research.

[17] Niedziela-Majka A, Chesnik MA, Tomko EJ, Lohman TM (2007). *Bacillus stearothermophilus* PcrA monomer Is a single-stranded DNA translocase but not a processive helicase in vitro.. Journal of Biological Chemistry.

[18] Dillingham MS, Wigley DB, Webb MR (2000). Demonstration of unidirectional single-stranded DNA translocation by PcrA helicase: measurement of step size and translocation speed.. Biochemistry.

[19] Slatter AF, Thomas CD, Webb MR (2009). PcrA helicase tightly couples ATP hydrolysis to unwinding double-stranded DNA, modulated by the replication initiator protein, RepD.. Biochemistry.

[20] Dillingham MS, Soultanas P, Wiley P, Webb MR, Wigley DB (2001). Defining the roles of individual residues in the single-stranded DNA binding site of PcrA helicase.. Proceedings of the National Academy of Science USA.

[21] Forgacs E, Cartwright S, Kovacs M, Sakamoto T, Sellers J (2006). Kinetic mechanism of myosinV-S1 using a novel fluorescent ATP analogue.. Biochemistry.

[22] Henn A, Cao W, Hackney DD, De La Cruz EM (2008). The ATPase cycle mechanism of the DEAD-box rRNA helicase, DbpA.. Journal of Molecular Biology.

[23] Toseland CP, Martinez-Senac MM, Slatter AF, Webb MR (2009). The ATPase cycle of PcrA helicase and its coupling to translocation on DNA.. Journal of Molecular Biology.

[24] Hsieh J, Moore KJM, Lohman TM (1999). A two-site kinetic mechanism for ATP binding and hydrolysis by *E. coli* Rep helicase dimer bound to a single-stranded oligodeoxynucleotide.. Journal of Molecular Biology.

[25] Moore KJ, Lohman TM (1994). Kinetic mechanism of adenine nucleotide binding to and hydrolysis by the *Escherichia coli* Rep monomer. 2. Application of a kinetic competition approach.. Biochemistry.

[26] Soultanas P, Dillingham MS, Velankar SS, Wigley DB (1999). DNA binding mediates conformational changes and metal ion coordination in the active site of PcrA helicase.. Journal of Molecular Biology.

[27] Korolev S, Hsieh J, Gauss GH, Lohman TM, Waksman G (1997). Major domain swiveling revealed by the crystal structures of complexes of *E. coli* Rep helicase bound to single-stranded DNA and ADP.. Cell.

[28] Lee JY, Yang W (2006). UvrD helicase unwinds DNA one base pair at a time by a two-part power stroke.. Cell.

[29] Velankar SS, Soultanas P, Dillingham MS, Subramanya HS, Wigley DB (1999). Crystal structures of complexes of PcrA DNA helicase with a DNA substrate indicate an inchworm mechanism.. Cell.

[30] Dillingham MS, Wigley DB, Webb MR (2002). Direct measurement of single stranded DNA translocation by PcrA helicase using the fluorescent base analogue 2-aminopurine.. Biochemistry.

[31] Henn A, Cao W, Licciardello N, Heitkamp SE, Hackney DD (2010). Pathway of ATP utilization and duplex rRNA unwinding by the DEAD-box helicase, DbpA.. Proceedings of the National Academy of Sciences.

[32] Webb MR, Johnson KA (2003). A fluorescent sensor to assay inorganic phosphate.. Kinetic Analysis of Macromolecules: a Practical Approach.

[33] Brune M, Hunter JL, Howell SA, Martin SR, Hazlett TL (1998). Mechanism of inorganic phosphate interaction with phosphate binding protein from *Escherichia coli*.. Biochemistry.

[34] Webb MR, Trentham DR (1981). The mechanism of ATP hydrolysis catalyzed by myosin and actomyosin, using rapid reaction techniques to study oxygen exchange.. Journal of Biological Chemistry.

[35] Jameson DM, Eccleston JF (1997). Fluorescent nucleotide analogs: synthesis and applications.. Methods in Enzymology.

[36] Toseland CP, Webb MR (2011). Fluorescent nucleotides for single molecule enzymology.. Methods in Molecular Biology.

[37] Hibberd MG, Webb MR, Goldman YE, Trentham DR (1985). Oxygen exchange between phosphate and water accompanies calcium-regulated ATPase activity of skinned fibers from rabbit skeletal muscle.. Journal of Biological Chemistry.

[38] Nixon AE, Hunter JL, Bonifacio G, Eccleston JF, Webb MR (1998). Purine nucleoside phosphorylase: its use in a spectroscopic assay for inorganic phosphate and to remove inorganic phosphate with the aid of phosphodeoxyribomutase.. Analytical Biochemistry.

[39] Leatherbarrow RJ (2007). GraFit Version 6, Erithacus Software Ltd..

[40] Johnson KA, Simpson ZB, Blom T (2009). Global Kinetic Explorer: A new computer program for dynamic simulation and fitting of kinetic data.. Analytical Biochemistry.

