# Supplementary figures and images for: ATPase Cycle and DNA Unwinding Kinetics of RecG Helicase
Source: PLoS One. 2012 Jun 6;7(6):e38270. doi: 10.1371/journal.pone.0038270 (PMC3368886; doi:10.1371/journal.pone.0038270)

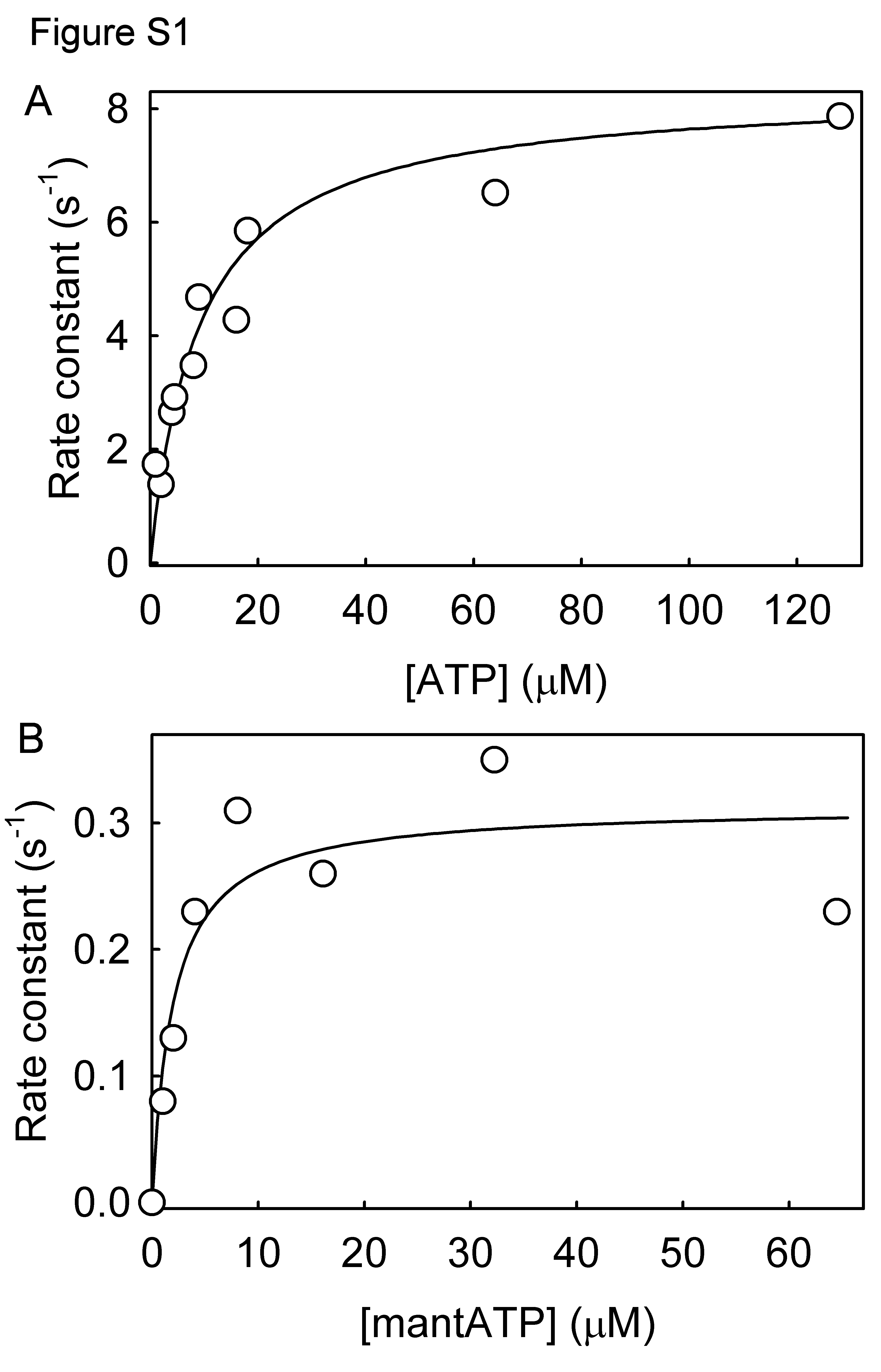

Supplement: Figure S1 — Steady state ATPase activity of RecG. The measurements were carried out at 20°C with solution conditions as described in Materials and Methods with 10 nM RecG, 500 nM DNA Junction (A40:B40), 10 µM MDCC-PBP and triphosphate nucleotide at the concentrations shown. (A) Steady-state measurements for ATP. The lines are best fits to the Michaelis-Menten equation and give a K m of 9 (±2) µM and a k cat of 8.3 (±1.7) s−1. (B) Steady-state measurements for mantATP. The best fit gives a K m of 1.9 (±0.6) µM and a k cat of 0.3 (±0.1) s−1. (TIF) [file pone.0038270.s001.tif]

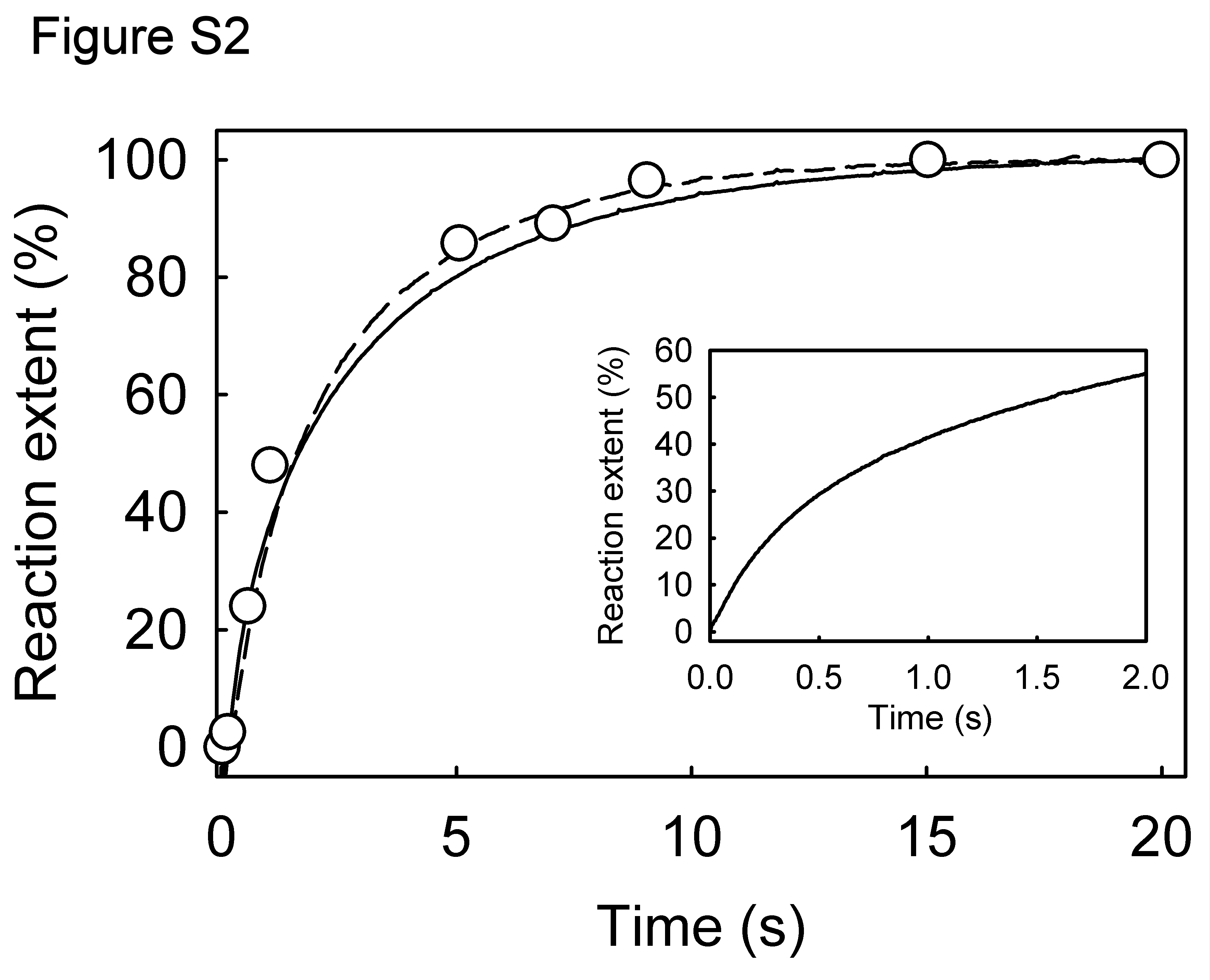

Supplement: Figure S2 — Kinetic measurements of mantATP with excess of RecG and a Three-strand DNA Junction. The concentrations for all experiments were 0.5 µM mantATP, 2.5 µM RecG, 5 µM DNA Junction (A40:B40:C19) and 10 µM MDCC-PBP (for Pi measurement). All measurements were carried out under the conditions of Figure 4. Time course of mant fluorescence (continuous line), mantADP formation (circles) and Pi release (dashed line). The insert shows the initial change in mant fluorescence. (TIF) [file pone.0038270.s002.tif]

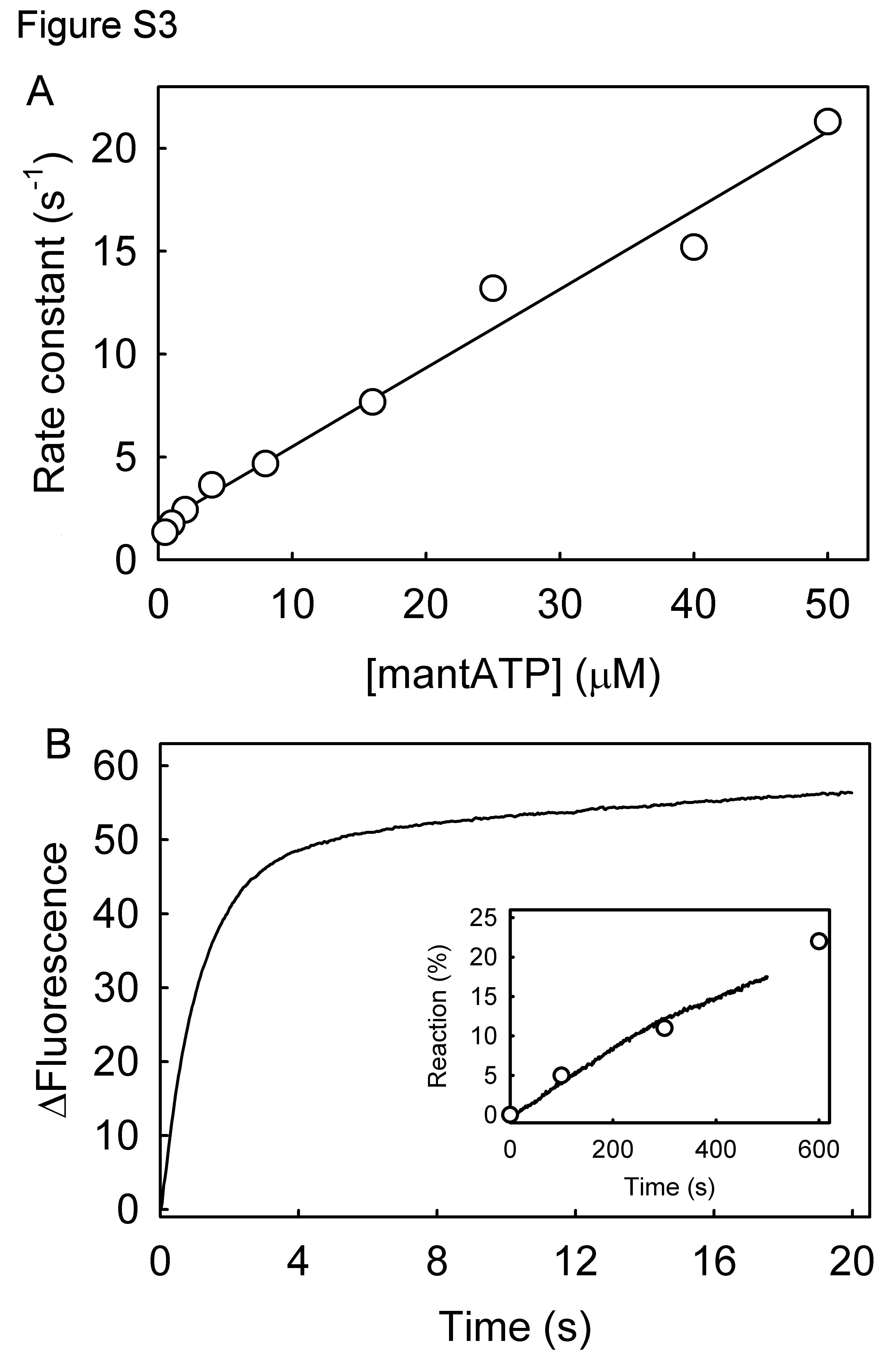

Supplement: Figure S3 — Kinetic measurements in the absence of DNA. (A) Association kinetics with mantATP. Dependence of the observed rate constants on mantATP concentration from mixing 0.5 µM RecG with excess mantATP under the same conditions as Figure 3. Points shown are typically an average of three individual measurements. The best linear fit gives a slope of 0.4 (±0.15) µM−1 s−1 and intercept 1.7 (±0.6) s−1. (B) Fluorescence trace upon mixing 2.5 µM RecG with 0.5 µM mantATP under the conditions of Figure 4. The trace was fitted by an exponential and a slope giving rates of 0.42 (±0.12) s−1 for the former. Note that as the fluorescence slowly increases beyond the time course, it was not possible to calibrate the ordinate. Inset: Hydrolysis and Pi release for these conditions. The circles represent single time points for a quenched-flow measurement of mantADP formation. Pi release kinetics (continuous line) were measured for the same mixture containing 10 µM MDCC-PBP. (TIF) [file pone.0038270.s003.tif]
