# Supplementary material for: ATPase Cycle and DNA Unwinding Kinetics of RecG Helicase
Source: PLoS One. 2012 Jun 6;7(6):e38270. doi: 10.1371/journal.pone.0038270 (PMC3368886; doi:10.1371/journal.pone.0038270)
Supplement: Table S1 — Oligonucleotide sequences. They are all are written 5′ to 3′. The E19 oligonucleotide is complementary to the B40 oligonucleotide and was used to create 4 strand non-complementary junctions. (PDF) [file pone.0038270.s004.pdf]

Table S1. Oligonucleotide sequences. They are all are written 5′ to 3′. The E19 oligonucleotide is complementary to the B40 oligonucleotide and is used to create 4 strand non-complementary junctions.

A30	GTA GTG CTC GTC TGG CTC TGG ATT ACC CGC
A35	TGT TCG TAG TGC TCG TCT GGC TCT GGA TTA CCC GC
A40	TTA GTT GTT CGT AGT GCT CGT CTG GCT CTG GAT TAC CCG C
A50	CGA CAT CAG TTT AGT TGT TCG TAG TGC TCG TCT GGC TCT GGA TTA CCC GC
A60	CCG TAT CTA TCG ACA TCA GTT TAG TTG TTC GTA GTG CTC GTC TGG CTC TGG ATT ACC CGC
C9	GAG CAC TAC
C14	GAG CAC TAC GAA CA
C19	GAG CAC TAC GAA CAA CTA A
C29	GAG CAC TAC GAA CAA CTA AAC TGA TGT CG
C39	GAG CAC TAC GAA CAA CTA AAC TGA TGT CGA TAG ATA CGG
B40	GCG GGT AAT CCA GAG CCA GAA TCA TAG CCA TAA CAC GAT C
B´30	GCG GGT AAT CCA GAG CCA GAC GAG CAC TAC
B´35	GCG GGT AAT CCA GAG CCA GAC GAG CAC TAC GAA CA
B´40	GCG GGT AAT CCA GAG CCA GAC GAG CAC TAC GAA CAA CTA A
B´50	GCG GGT AAT CCA GAG CCA GAC GAG CAC TAC GAA CAA CTA AAC TGA TGT CG
B´60	GCG GGT AAT CCA GAG CCA GAC GAG CAC TAC GAA CAA CTA AAC TGA TGT CGA TAG ATA CGG
D9	GTA GTG CTC
D14	TGT TCG TAG TGC TC
D19	TTA GTT GTT CGT AGT GCT C
D29	CGA CAT CAG TTT AGT TGT TCG TAG TGC TC
D39	CCG TAT CTA TCG ACA TCA GTT TAG TTG TTC GTA GTG CTC
E19	GAT CGT GTT ATG GCT ATG A
