# Supplementary material for: ATPase Cycle and DNA Unwinding Kinetics of RecG Helicase
Source: PLoS One. 2012 Jun 6;7(6):e38270. doi: 10.1371/journal.pone.0038270 (PMC3368886; doi:10.1371/journal.pone.0038270)
Supplement: Table S2 — Oxygen exchange during ATP or mantATP hydrolysis by RecG: distributions of oxygen-18 in the Pi product. Experiments were performed as described in Materials and Methods. The data were corrected for isotopic enrichment of the starting nucleotide, 93% for (γ-18O3)mantATP and 98% for (γ-18O3) ATP. The table shows the distributions of isotope in the product Pi after unlabeled Pi was subtracted (along with natural abundance in the (18O1)Pi position), likely to be mainly contamination. Each distribution is the average of three mass spectral assays. Thus the distributions are for the product Pi as though the starting enrichment was 100%. In all cases there was very little oxygen exchange as shown by the large peak of (18O3)Pi, in which all three γ-oxygens of ATP are retained. The distributions were then used to compute the ratio of rate constants for Pi release (k +3) and on-enzyme ATP resynthesis (k −2) as in Figure 2 [14], [37]. The Pi from ATP hydrolysis consistently gave an abnormal distribution (as shown by higher percentage of (18O1)Pi, suggesting the possibility of a second minor activity. However, the analysis assumed a single pathway. (PDF) [file pone.0038270.s005.pdf]

Table S2: Oxygen exchange during ATP or mantATP hydrolysis by RecG: distributions of oxygen-18 in the P_i product.

Experiments were performed as described in Materials and Methods. The data were corrected for isotopic enrichment of the starting nucleotide, 93% for (γ-$^{18}O_3$)mantATP and 98% for (γ-$^{18}O_3$) ATP. The table shows the distributions of isotope in the product P_i after unlabeled P_i was subtracted (along with natural abundance in the ($^{18}O_1$)P_i position), likely to be mainly contamination. Each distribution is the average of three mass spectral assays. Thus the distributions are for the product P_i as though the starting enrichment was 100%. In all cases there was very little oxygen exchange as shown by the large peak of ($^{18}O_3$)P_i, in which all three γ-oxygens of ATP are retained. The distributions were then used to compute the ratio of rate constants for P_i release (k_{+3}) and on-enzyme ATP resynthesis (k_{-2}) as in Figure 2 [14,36]. The P_i from ATP hydrolysis consistently gave an abnormal distribution (as shown by higher percentage of ($^{18}O_1$)P_i, suggesting the possibility of a second minor activity. However, the analysis assumed a single pathway.

**Nucleotide**	**DNA present**	**($^{18}O_1$)P_i (%)**	**($^{18}O_2$)P_i (%)**	**($^{18}O_3$)P_i (%)**	***Best fit distribution*** **($^{18}O_1$)P_i:($^{18}O_2$)P_i:($^{18}O_3$)P_i**	**k_{+3}/ k_{-2}**
mantATP	+	0.4	7.5	92.1	0.4:7.6:92.0	8.7
mantATP	-	0.4	7.0	92.6	0.4:7.0:92.7	9.5
ATP	+	2.8	2.3	94.9	0.1:3.9:96.0	18
ATP	-	6.1	4.0	89.9	0.4:7.3:92.3	9.0
