# Supplementary material for: ATPase Cycle and DNA Unwinding Kinetics of RecG Helicase
Source: PLoS One. 2012 Jun 6;7(6):e38270. doi: 10.1371/journal.pone.0038270 (PMC3368886; doi:10.1371/journal.pone.0038270)
Supplement: Table S3 — Kinetic parameters describing RecG binding to various model substrates under pseudo-first order conditions. The junctions are made up from the individual oligonucleotides, as defined in Table S1, but with a Cy3 at the 5′-end of the single strand part of the junction. Binding kinetics were measured with conditions as described in the Materials and Methods at 10 nM RcG and varying the junction concentration in the range 100 nM to 1000 nM. After fitting the fluorescence curves to single exponentials, the observed rate constants were plotted as a function of DNA concentration. Assuming a single step binding, the rate constants were obtained and their ratio gave the K d values. Some experiments were done in the presence of 200 µM adenosine nucleotide. The major differences are in the association rate constant: the dissociation varies little. (PDF) [file pone.0038270.s006.pdf]

Table S3. Kinetic parameters describing RecG binding to various model substrates under pseudo-first order conditions.

The junctions are made up from the individual oligonucleotides, as defined in Table S1, but with a Cy3 at the 5′-end of the single strand part of the junction. Binding kinetics were measured with conditions as described in the Materials and Methods at 10 nM RcG and varying the junction concentration in the range 100 nM to 1000 nM. After fitting the fluorescence curves to single exponentials, the observed rate constants were plotted as a function of DNA concentration. Assuming a single step binding, the rate constants were obtained and their ratio gave the K_d values. Some experiments were done in the presence of 200 μM adenosine nucleotide. The major differences are in the association rate constant: the dissociation varies little.

Strands	Complementary	ATP/ADP	k_{+1} (μM^{-1} s^{-1})	k_{-1} (s^{-1})	K_d (nM)
A40:B40	no	no	172 (±23)	39 (±6)	226
A40:B40	no	ATP	462 (±44)	26 (±7)	57
A40:B40:C19:E19	no	no	86 (±12)	55 (±3)	642
A40:B′40:C19:D19	yes	no	75 (±7)	59 (±2)	788
A40:B′40:C19:D19	yes	ATP	348 (±35)	28 (±5)	80
A40:B′40:C19:D19	yes	ADP	261 (±17)	43 (±3)	163
